# Author’s reply

**DOI:** 10.4103/0972-2327.70889

**Published:** 2010

**Authors:** M. V. Padma Srivastava

**Affiliations:** Department of Neurology, AIIMS, New Delhi – 110 029, India

I thank the author for the very relevant observations made in the letter.

1. Regarding the first comment, it is true that the Clopidogrel versus Aspirin in Patients at Risk of Ischemic Events (CAPRIE) study showed a relative risk reduction of 8.7% in favor of clopidogrel, which certainly is not an ‘immense’ benefit over and above that with aspirin. I apologize for the typing error.
2. Regarding the second comment, the concepts of antiplatelet ‘resistance’ and ‘failure’ are still evolving. I agree that the overall risk reduction with aspirin is so small that while on aspirin, recurrence is very possible. However, all things being equal, if on an adequate prophylaxis (with whatever is possible, including risk factor reduction, etc.), a patient continues to have recurrent events, it does call for a change in strategy rather than persisting with the same course of action.
3. Regarding the third comment, I do not think that we have a final word on ‘any’ aspect of antiplatelet therapy as of today. One of the major criticisms of the European Stroke prevention Study (ESPS) has been the choice of aspirin dosage. In (European and Australasian Stroke Prevention in Reversible Ischemia Trial (ESPRIT) the dosage did vary and some received up to 325 mg of aspirin. In Management of Atherothrombosis with Clopidogrel in High risk patients with recent transient ischemic attacks (TIAs) or ischemic stroke (IS) (MATCH) the dosage of aspirin was 75 mg, which is in the ‘accepted’ range for prophylaxis and was compared with clopidogrel. This review is essentially an attempt to point out some aspects of the existing controversies, confusion, and a definite need for further evidence in many areas of stroke prophylaxis.

